# New Remedies: With Formulæ for Their Preparation and Administration

**Published:** 1857-03

**Authors:** 


ART. VIII.—*New Remedies: with Formulæ for their Preparation and Administration.* By ROBLEY DUNGLISON, M. D., Professor of the Institutes of Medicine, etc., in the Jefferson Medical College of Philadelphia. Seventh Edition, with numerous additions. Prodesse quam conspici. Philadelphia: BLANCHARD & LEA. 1856.

The profession at large are familiar with the merits of Dunglison's New Remedies. Important additions to the materia medica are made with such rapidity, that they necessarily go out to the profession before they can be adopted in the decennial conventions for the revision of the pharmacopæia. In the meantime, it is important that the profession should be possessed of these new remedies, and should have some source to which to look for information as to their sensible properties, tests of purity, and their therapeutical value. Dr. Dunglison has supplied this want, and the high estimation in which his work is held, is evinced by the fact that this is the seventh edition published.

It is useless for us to enlarge upon the qualities of a book so well known. The time for criticism upon it has passed. But we may introduce to our readers some of the additional new remedies, described for the first time in this edition, and so furnish an article of some practical value. The number is somewhat large, but not, except in some instances, of very great interest. We take them up alphabetically.

1. *Apiol.* This is a yellowish, oily liquid, obtained from the seeds of the common parsley. It has a piquant, acrid taste, with a peculiar odor. It has been used with success in the treatment of intermittents in Europe. The medium dose is about 15 grs. It produces an intoxication like that of quinine, in 30 to 40 grain doses. It has succeeded in 86 cases out of 100 in the intermittents of Europe. Its effects are closely analogous to those of quinine.

2. *Caffein*, the active principle of coffee, is now pretty largely used in sick headaches and idiopathic hemicrania. One grain doses are taken every hour. The extract of coffee is a cheap and valuable substitute.

3. *Chloride of Iron* is mentioned in connection with erysipelas, scarlatina, and as an injection in the cure of aneurism. Three or four drops of the concentrated solution of the perchloride are injected into the diseased artery. It produces a firm, solid clot. We may add that we have used the diluted muriated tincture as an injection in profuse epistaxis, with very gratifying results.

4. *Chloride of Sodium.* The use of common salt in intermittents, is referred to. It is a philosophical remedy, and we have seen good effects follow its use.

5. *Rennet*, in the treatment of diabetes, is likely to assume an important place. The testimony in its favor is very considerable.

Quite a number of other articles are given, and, aside from these additions, the author has considerably expanded his notes on remedies introduced in previous editions. The book will be found worthy of purchase even by those who have the previous editions.

For sale by PHINNEY & Co.

